# Cfap91-Dependent Stability of the RS2 and RS3 Base Proteins and Adjacent Inner Dynein Arms in *Tetrahymena* Cilia

**DOI:** 10.3390/cells11244048

**Published:** 2022-12-14

**Authors:** Marta Bicka, Ewa Joachimiak, Paulina Urbanska, Anna Osinka, Anna Konopka, Ewa Bulska, Dorota Wloga

**Affiliations:** 1 Faculty of Chemistry, University of Warsaw, 1 Pasteur Street, 02-093 Warsaw, Poland; 2 Laboratory of Cytoskeleton and Cilia Biology, Nencki Institute of Experimental Biology of Polish Academy of Sciences, 3 Pasteur Street, 02-093 Warsaw, Poland; 3 Faculty of Chemistry, Biological and Chemical Research Centre, University of Warsaw, 101 Żwirki i Wigury Street, 02-089 Warsaw, Poland; 4 Łukasiewicz Research Network—PORT Polish Center for Technology Development, 147 Stablowicka Street, 54-066 Wrocław, Poland

**Keywords:** cilia, axoneme, radial spoke, CFAP91, *Tetrahymena*

## Abstract

Motile cilia and eukaryotic flagella are specific cell protrusions that are conserved from protists to humans. They are supported by a skeleton composed of uniquely organized microtubules—nine peripheral doublets and two central singlets (9 × 2 + 2). Microtubules also serve as docking sites for periodically distributed multiprotein ciliary complexes. Radial spokes, the T-shaped ciliary complexes, repeat along the outer doublets as triplets and transduce the regulatory signals from the cilium center to the outer doublet-docked dynein arms. Using the genetic, proteomic, and microscopic approaches, we have shown that lack of *Tetrahymena* Cfap91 protein affects stable docking/positioning of the radial spoke RS3 and the base of RS2, and adjacent inner dynein arms, possibly due to the ability of Cfap91 to interact with a molecular ruler protein, Ccdc39. The localization studies confirmed that the level of RS3-specific proteins, Cfap61 and Cfap251, as well as RS2-associated Cfap206, are significantly diminished in *Tetrahymena* CFAP91-KO cells. Cilia of *Tetrahymena* cells with knocked-out *CFAP91* beat in an uncoordinated manner and their beating frequency is dramatically reduced. Consequently, CFAP91-KO cells swam about a hundred times slower than wild-type cells. We concluded that *Tetrahymena* Cfap91 localizes at the base of radial spokes RS2 and RS3 and likely plays a role in the radial spoke(s) positioning and stability.

## 1. Introduction

Motile cilia and homologous flagella are motor proteins-containing autonomously- moving structures that are conserved in organisms from most eukaryotic kingdoms. Their activity enables the movement of single-celled and some multicellular organisms. In most Metazoa, motile cilia play an important role during development and in proper organ/body function. In humans, the movement of cilia assembled by multiciliated epithelial cells lining internal passages, drives the circulation of the cerebrospinal fluid in brain ventricles [1] and the removal of inhaled bacteria or particles from the airways [2], in females, supports the transport of oocytes in the oviduct [3], and in males, counters sperm cell aggregation in the efferent duct while a single flagellum propels sperm cells [4,5]. During early development, the rotatory-like movement of nodal cilia contributes to the establishment of the left–right asymmetry of visceral body organs [6,7]. Lack or dysfunction of motile cilia causes primary ciliary dyskinesia (PCD) and male infertility [8].

**Figure 1:**
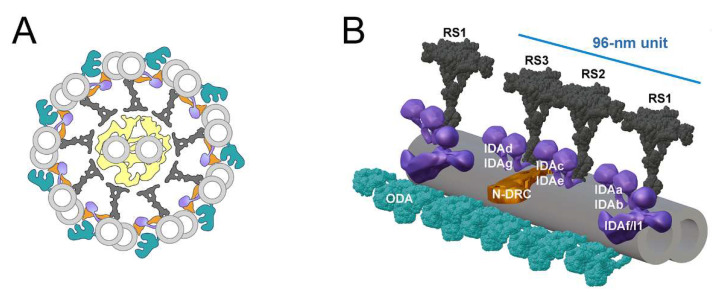
Schematic representation of the motile cilium structure. (**A**) Cilium cross-section. Two central microtubules (grey), together with associated complexes, a connecting bridge and projections (yellow), form a central apparatus. Main outer doublets (grey) complexes: outer dynein arms (ODAs, greenish), inner dynein arms (IDAs, purple), radial spokes (RSs, dark grey), nexin-dynein regulatory complex (N-DRC, orange); (**B**) Fragment of the outer doublet showing organization of ciliary complexes within a 96-nm outer doublet axonemal unit [9,10]. ODA (7K5B, PDB DOI: 10.2210/pdb7K5B/pdb [11]) and RS (7JTK, PDB DOI: 10.2210/pdb7JTK/pdb [12]) structures were obtained from a Protein Data Bank (https://www.rcsb.org (accessed on 20 November 2022)). Other ciliary complexes were designed using Adobe Photoshop and Blender programs.

Motile cilia are supported by uniquely organized microtubules—nine peripheral microtubular doublets and two central microtubules. Doublet microtubules are extensions of two out of three more inner microtubules of the basal body, a structure that anchors cilium to the cell surface. Both central and peripheral microtubules serve as a scaffold for periodically attached multiprotein ciliary complexes. The arrangement of ciliary complexes along outer doublets sets down the so-called 96-nm axonemal unit (Figure 1).

Each axonemal unit contains: (i) three T-shaped radial spokes, extending in the direction of the central apparatus, (ii) a nexin-dynein regulatory complex (N-DRC) coordinating/regulating other complexes and connecting adjacent outer doublets, and (iii) two types of motor protein-containing dynein arms, seven inner (IDA) and four outer (ODA). In contrast to ODAs whose structure and protein composition are similar, IDAs are either two-headed (IDA f/I1) or single-headed (IDA a, b, c, e, d, and g) and contain an arm-specific dynein heavy chain. The single-headed IDAs are docked by their tail part onto the base of the radial spokes: IDA a and b at RS1, IDA c and e at RS2 and IDA d and g at RS3 (for review [9]).

Immunoprecipitation studies using anti-calmodulin (CaM) antibodies and flagellar proteins from *Chlamydomonas pf14,* an RS1 and RS2 spoke-less mutant led to the identification of so-called calmodulin and spoke-associated complex (CSC) composed of FAP61, FAP91, and FAP251 [13]. Further analyses showed that components of CSC co-purify with radial spoke proteins and are indispensable for proper cilia and flagella beating [13,14].

In *Chlamydomonas*, amiRNA-based knock-down of *fap91* or *fap61* reduced cells’ swimming rate. The mutants’ flagella beat with only a slightly reduced frequency but often paused at the end of the recovery stroke and failed to perform a full effective stroke. Consequently, mutants’ flagella lost synchrony [14]. In *Chlamydomonas* flagella, only two spokes, RS1 and RS2, are full-length structures while RS3 is in the form of a short knob [15,16,17]. The ultrastructural studies (cryo-ET) revealed that, in both *fap61* and *fap91* mutants, RS2 and IDA e were frequently missing while the RS3 structure was reduced [14,18].

In a parasitic flagellated *T. brucei*, the RNAi-based knock-down of *TbCFAP91* [19] or *TbCFAP251* [20] also significantly reduced cell motility. Regrettably, the radial spoke structure was not investigated in these mutants.

In a ciliate *Tetrahymena thermophila,* deletion of Cfap61 or Cfap251 also slowed down the cells’ swimming rate [21]. Axonemes of the *CFAP61* and *CFAP251* knock-out mutants lacked either the entire RS3, or more frequently, only a specific region of RS3: a part of the RS3 stalk in CFAP61-KO mutant or a part or the entire arch-like structure at the RS3 base in the CFAP251-KO cell [21].

Mutations in genes encoding CSC components also affect proper cilia/flagella functioning in vertebrates. In humans, mutations in genes encoding CFAP61 [22,23,24], CFAP91/AAT1/MAATS1/C3orf15 [19], or CFAP251/WDR66 [20,25,26,27] cause male infertility. This agrees with the gene expression studies in mice [23] and human tissues [28,29], showing that *CFAP61*, *CFAP91*, and *CFAP251* are highly expressed in testis.

The up-to-date studies of *Chlamydomonas* and *Tetrahymena* mutants indicate that FAP61 and FAP251 are, in fact, radial spoke proteins [18,21]. Much less is known about the precise intraciliary localization of FAP91 protein. Based on atomic models, it was recently proposed that *Chlamydomonas* FAP91 forms a triple-helix with molecular ruler proteins, FAP59/CCDC39 and FAP172/CCDC40, and functions as an RS2 adaptor [12].

The bioinformatics analysis of FAP91 orthologs revealed that the middle and carboxy-terminal fragments of *Chlamydomonas* FAP91 are very divergent (Figure S1). Moreover, *Chlamydomonas* flagella have an atypical, strongly reduced RS3 structure. Therefore, we investigated the localization and role of Cfap91 using *Tetrahymena thermophila* as a model. We show that the presence of Cfap91 is crucial for the stability of the RS3 and the base of RS2 as well as RS2 and RS3 base-docked IDAs, and that the lack of Cfap91 dramatically affects cilia beating frequency and cell motility.

## 2. Methods

### 2.1. Tetrahymena Cells Strains and Culture Conditions

Wild-type and motile *Tetrahymena* mutants were grown in a standard SPP medium [30]. Mutant cells with reduced cell motility were cultured in 12-well plates in a rich MEPP medium with shaking (110 rpm) [31]. Media were supplemented with an antibiotic-antimycotic mix (Sigma-Aldrich, St. Louis, MO, USA) at 1:100 (SPP) or 1:50 (MEPP).

### 2.2. Gene Knock-Outs and Knock-Ins

*Tetrahymena* mutants with knocked-out *CFAP206* [32], *CFAP61,* or *CFAP251* [21] genes were described previously. To delete *CFAP91* using a germ-line approach [33,34], two fragments of the gene were amplified by PCR with the addition of restriction sites using high fidelity polymerase, and subsequently cloned into pNeo4 plasmid [35] digested with the same enzymes. The 1.3 kb fragment amplified using 5′AATT GGGCCC GAGCTC CAGAATTATTATAGGAAGAAGTTAAAGG 3′ and 5′AATT CCCGGG TATTGGAGCAAATAAAGTTTCTCAT 3′ primers encompassed 80 bp of the 5′UTR and 1.2 kb of the open reading frame. The second fragment positioned 800 bp downstream from the first one was amplified using 5′AATT CTGCAG GGAGTACAGAGGTCCTAATG 3′ and 5′ATAT CCGCGG GGTACC TCATATATCTTTGGCAGAGTACATA 3′ primers and encompassed 0.96 kb of the coding region and 0.26 kb of 3′UTR. In the rescue experiment, CFAP91-KO cells were transformed with a plasmid enabling the expression of the Cfap91-HA fusion protein [36] in the non-essential *BTU1* locus.

To express C-terminally 3HA-tagged proteins in their native loci, the coding region of 2V5 in pFAP44-2V5-pPur plasmid [37] was replaced by a fragment encoding 3HA tag. Approximately 1 kb fragments of the open reading frame without a stop codon and 3′ untranslated region (3′UTR) were amplified from the genomic DNA with the addition of unique restriction sites (primers in Table S1), digested with restriction endonucleases, and cloned into a pFAP44-3HA-pPur plasmid with pPur cassette [38]. About 10 μg of the final plasmids were digested with *MluI* and *XhoI* and used for biolistic transformation. Transformants were selected for 3–4 days at 30°C on SPP or MEPP with 1.5 μg/mL CdCl_2_ and 200 μg/mL puromycin (BioShop Canada Inc., Burlington, ON, Canada) and grown under puromycin selection to promote phenotypic assortment.

Preparation of *Tetrahymena* cells overexpressing GFP-tagged Cfap61 and Cfap251 proteins has been described before [21]. To engineer cells overexpressing Cfap91 as GFP fusion protein, the *CFAP91* coding region was cloned into RSP4A-GFP plasmid [39]. To overexpress calmodulin or Ccdc39 with N-terminal HA tag, the coding region was amplified with the addition of MluI and BamHI sites and cloned into a modified version of the vector enabling incorporation into the *BTU1* locus and selection using neo2 cassette [39]. The primers used are listed in Table S1.

### 2.3. Phenotypic Analyses

To measure the cell swimming rate, cells were grown to the density of 2–2.5 × 10^5^ cells/mL, diluted to the density of 10^4^ cells/mL with a medium pre-warmed to 30 °C. Approximately 700 µL of cells were placed on the bottom of the well of a 24-well plate and swimming cells were recorded at room temperature using a Zeiss Discovery V8 Stereo microscope (Zeiss, Oberkochen, Germany) equipped with Zeiss Plans 10× FWD 81 mm objective and Axiocam 506 camera and ZEN2 (blue edition) software. The lengths of the swimming paths were measured using ImageJ software.

To analyze cilia beating, cells from an overnight culture were diluted to the density of 10^5^ cells/mL and incubated at room temperature for three hours. Next, approximately a 30 µL drop of cells in an SPP medium or SPP medium mixed 1:1 with 1.5% methylcellulose in 10 mM Tris-HCl buffer, pH 7.4 was placed between two pieces of adhesive tape fixed on the glass slide and covered with a coverslip. At least 10 cells were recorded using the Phantom Miro C110 high-speed camera (Vision Research, Wayne, NJ, USA) mounted on an AXIO Imager M2 microscope (Zeiss, Germany) using either a 40× oil immersion lens (analyses of cilia beating frequency) or a 63× oil immersion lens (numerical aperture 1.4, analyses of ciliary waveform). Videos were recorded at 900 frames/s. For the cilia beating frequency, 450 frames were aligned and analyzed using ImageJ 1.52p (http://imagej.nih.gov/ij, accessed on 20 November 2022). For waveform analyses, cells were aligned using ImageJ and analyzed in Adobe PhotoShop 21.2.3 (https://www.adobe.com/pl/creativecloud/plans.html?promoid=B16P3VKM&mv=otherplans.html?promoid=B16P3VKM&mv=other, accessed on 20 November 2022) to show the position of the cilium in subsequent frames.

### 2.4. Immunofluorescence and Ultrastructural Studies

For immunofluorescence analyses, a 10 µL drop of cells [21] was placed on a coverslip and cells were fixed with 15 µL of a mixture of 0.5% Triton-X-100 and 8% PFA (1:1) in a PHEM buffer. After drying and blocking the unspecific antibodies binding with 3%BSA/PBS, cells were stained with anti-HA antibody (BioLegend, San Diego, CA, USA) diluted 1:300 and left overnight at 4 °C. To visualize cilia, including their tip, cells (10 µL) were first permeabilized with an equal volume of 0.5% Triton-X-100/PHEM buffer for 40 s, fixed with 20 µL of 8% PFA/PHEM, and after blocking, stained with a mix of polyG antibodies [40], diluted 1:2000 and anti-α-tubulin 12G10 antibodies (Developmental Studies Hybridoma Bank, University of Iowa, Iowa City, IA, USA) diluted 1:200. After washing with PBS (3 × 5 min) samples were stained for 1.5 h at RT with the secondary antibodies anti-mouse or anti-rabbit IgG, conjugated with either Alexa-488 or Alexa-555 (Invitrogen, Eugene, OR, USA) diluted 1:300. After washing the coverslips were mounted in Fluoromount-G (Southern Biotech., Birmingham, AL, USA). Cells were recorded using either a Zeiss LSM780 (Carl Zeiss Jena, Germany) or a Leica TCS SP8 (Leica Microsystems, Wetzlar, Germany) confocal microscope.

For ultrastructural studies, cells were fixed and analyzed as described [21]. Briefly, cells from a 5 mL mid-log phase culture were spun down, washed with 10 mM Tris-HCl buffer (pH 7.4), fixed on ice for one hour first with 2% glutaraldehyde in 0.1 M cacodylate buffer, pH 7.2, and next, for another hour in the same solution with the addition of tannic acid (0.01% final). After washing with 0.1 M cacodylate buffer, samples were fixed (1 h) with 1% osmium tetroxide. After washing with water and embedding fixed cells in 2% low-melting agarose, 2–3 mm pieces of agarose with cells were dehydrated and mounted in Durcupan ACM Fluka (Sigma-Aldrich, St. Louis, MO, USA). Approximately 40–50 nm thick sections were viewed using a JEM 1400 transmission electron microscope (JEOL Co, Tokyo, Japan) and cilia were recorded (magnification: 100,000–200,000) using an 11 Megapixel TEM Morada G2 camera (EMSIS GmbH, Münster, Germany).

### 2.5. Western Blot and 2D-Electrophoresis

Cilia were collected from 100 mL a mid-log phase culture as described [41]. Briefly, cells were collected by centrifugation, washed with 10 mM Tris-HCl buffer (pH 7.4), suspended in 40 mL of deciliation buffer (10 mM Tris-HCl, pH 7.4, 10 mM CaCl_2_, 50 mM sucrose with protease inhibitors (cOmplete, EDTA-free protease inhibitor cocktail, Roche Diagnostics GmbH, Mannheim, Germany), and deciliated by a pH shock. Deciliation was monitored under a microscope. Samples containing intact non-moving cells were spun down twice at 1680× *g* for 5 min to remove cell bodies and the supernatant was centrifuged at 23,300× *g* for 30 min to collect cilia. The ciliary pellet was suspended in 10 mM Tris-HCl buffer (pH 7.4) with protease inhibitors. The total protein concentration was measured with the Pierce™ BCA Protein Assay Kit (Thermo Scientific, Bartlesville, OK, USA).

For SDS-PAGE and Western blot analyses, 30 µg of ciliary proteins were loaded on the gel. HA-tagged fusion proteins were detected using anti-HA antibodies diluted 1:2000.

For 2D electrophoresis, 30 µg of ciliary proteins were cleaned using ReadyPrep 2-D Cleanup Kit (Bio-Rad, Laboratories, Herculers, CA, USA) and separated using 7 cm either 3–10 or 4–7 ReadyStrip^TM^ IPG Strips (Bio-Rad Laboratories, Herculers, CA, USA) and Protean IEF Cell (Bio-Rad Laboratories, Herculers, CA, USA) at 4000 V for 80,000 kVh at 20 °C. The HA-tagged fusion protein isoforms were detected, as described above.

### 2.6. Proximity Labeling (BioID) and Pull-Down Assays

The proximity-labeling assay [42] was performed as described in detail [39]. Briefly, wild-type cells (control) and cells expressing Cfap91-HA-BirA* were grown in 200 mL of SPP to the density 2.5 × 10^5^ cells/mL, spun down and suspended in 200 mL of 10 mM Tris-HCl buffer (pH 7.4). After 16–18 h, the biotin was added (50 µM final concentration) to the medium, and after 4 h of incubation at 30 °C, cells were deciliated and cilia were collected as described above. To remove the ciliary membrane, the ciliary pellet was suspended in axoneme stabilization buffer (20 mM potassium acetate, 5 mM MgSO_4_, 0.5 mM EDTA, 20 mM HEPES, pH 7.5 with protease inhibitors) supplied with 0.2% NP-40 and after 5 min the axonemes were spun down (30 min at 23,300× *g* at 4 °C) and suspended in a lysis buffer (50 mM Tris–HCl, pH 7.4, 0.4% SDS, 0.5 M NaCl, 1 mM DTT with protease inhibitors). After an hour, samples were spun down (8000× *g* at 4 °C). The collected supernatant was diluted with 50 mM Tris–HCl, pH 7.4 (1:3) and biotinylated proteins were bound overnight to streptavidin-coupled Dynabeads (Dynabeads M-280 Streptavidin, Thermo Fisher Scientific, Waltham, MA) at 4 °C. The collected proteins were analyzed by a Western blot and identified by mass spectrometry.

For pull-down analyses, cells overexpressing HA- or GFP- tagged proteins were grown to the mid-log phase in the SPP medium. The protein overexpression was induced by the addition of the cadmium chloride to the final concentration of 2.5 µg/mL. After 3–4 h of the induction, cells were collected by centrifugation, washed in 10 mM Tris-HCl buffer, pH 7.5, and solubilized in the two-times concentrated modified RIPA buffer (50 mM Tris-HCl, pH 7.5, 300 mM NaCl, 2% NP-40, 2% sodium deoxycholate, 10% glycerol with protease inhibitors). After spinning down at either 21,000× *g* or 100,000× *g* for 30 min at 4 °C, the supernatant was collected and diluted 1:4 with dilution buffer (25 mM Tris-HCl, pH 7.5, 150 mM NaCl, 5% glycerol with protease inhibitors). About 250–500 µg of proteins were used to absorb GFP-tagged proteins on anti-GFP-conjugated magnetic agarose beads (ChromoTek GFP-Trap^®^ Magnetic Agarose) according to the manufacturer’s instructions. After washing (5 × 5 min) with a Wash buffer (25 mM Tris-HCl, pH 7.5, 150 mM NaCl, 0.2% NP-40.5% glycerol with protease inhibitors) to remove unbound proteins, magnetic beads with absorbed GFP-tagged proteins were incubated with 250–500 µg proteins purified from cells expressing HA-tagged fusion protein. After an hour of incubation at room temperature, and washing (as above), proteins bound to the beads were solubilized in hot Laemmli buffer, loaded onto SDS-PAGE and analyzed by Western blot using anti-GFP (1:60,000, Abcam) and anti-HA antibodies (1:2000).

### 2.7. Total Label-Free Cilia Proteome Analyses

Cilia purified from 5 × 10^7^ cells were resuspended in the axoneme stabilization buffer (20 mM potassium acetate, 20 mM HEPES pH 7.6, 5 mM MgSO_4_, 0.5 mM EDTA) containing 0.2% NP-40 and incubated for 5 min on ice to remove the ciliary membrane. Next, axonemes were lysed with 1% SDS, 50 mM Tris–HCl, pH 7.4. The total protein concentration was measured with the Pierce™ BCA Protein Assay Kit (Thermo Scientific, Bartlesville, OK) following the manufacturer’s procedure, and 300 μg of proteins per sample were precipitated overnight with ReadyPrep 2-D Cleanup Kit (Bio-Rad Laboratories, USA). After incubation, samples were centrifuged for 15 min at 20,000× *g* and supernatants were discarded. In-solution protein digestion was performed as described [43] with minor modifications. Briefly, protein pellets were resuspended in 50 µL of 0.1% RapiGest in 500 mmol/L Triethylammonium bicarbonate buffer and reduced with 1,4-dithiothreitol (final concentration 10 mmol/L) for 45 min at 56 °C, then alkylated with iodoacetamide (final concentration 30 mmol/L) for 30 min. Proteins were enzymatically digested overnight with trypsin (Trypsin Gold, Mass Spectrometry Grade, Promega), weight ratio 100:1 protein to enzyme at 37 °C. The digestion reaction was quenched by the addition of 55% trifluoroacetic acid to the final concentration of 5%. After centrifugation for 30 min at 20,000× *g* at 4 °C, the supernatants were transferred to fresh Eppendorf tubes. Peptides were purified with Pierce™ Peptide Desalting Spin Columns (Thermo Scientific, Bartlesville, OK, USA) according to the manufacturer’s protocol, dried in a vacuum concentrator at room temperature, and resuspended in 100 μL of 5% acetonitrile and 0.1% formic acid. Next, samples were analyzed on the Evosep One system (Evosep Biosystems, Odense, Denmark) coupled to an Orbitrap Exploris 480 mass spectrometer (Thermo Fisher Scientific, Bremen, Germany) according to [44] with some modifications. Briefly, 1 µg of cleaved peptides was loaded onto Evotips C18 trap columns (Evosep Biosystems, Odense, Denmark). Evotips were activated with solvent B (0.1% formic acid in acetonitrile) followed by incubation with 2-propanol and equilibration with solvent A (0.1% formic acid in water). Cleaved peptides from each sample were bound to the Evotips solid phase and then washed with solvent A. Samples were separated at a flow rate of 500 nl/min for 88 min gradient on the EV1106 analytical column (Dr. Maisch C18 AQ, 1.9 µm beads, 150 µm ID, 15 cm long, Evosep Biosystems, Odense, Denmark). The capillary voltage in nano- ESI source was set to 2.1 kV and the capillary temperature was set to 275 °C. Positive ionization and DDA (data dependent acquisition) mode were applied to collect data.

The MS parameters were as follows: MS1: resolving power 60,000, AGC target 300%, and the m/z range of 300 to 1600. MS2: resolving power 15,000, normalized AGC target. Forty, of the most abundant precursors ions within an isolation window of 1.6 m/z were fragmented. The intensity threshold was retained at 5 × 10^3^. The HCD (higher energy collisional dissociation) mode with a normalized collision energy of 30% was applied for precursor ion fragmentation.

### 2.8. MS/MS Data Analysis

Protein identification was performed with FragPipe (v. 17.1) (Nesvilab, University of Michigan, Ann Arbor, MI). The raw data were processed to mzML format using ProteoWizard’s MSConvert (v. 3.0.1908) (Palo Alto, CA, USA). The *Tetrahymena thermophila* Uniprot database (canonical and isoform sequences; 27,027 entries;) was searched using the following search parameters: (i) Digestion enzyme-trypsin/P, (ii) precursor and fragment ion mass tolerance ±10.0 ppm and 0.6 Da, respectively, (iii) fixed modifications: carbamidomethyl (C), (iv) variable modifications: oxidation (M), deamidation (N), (Q), and, for BioID samples, and (v) biotinylation of lysine. The target-decoy approach with the reversed database was used to identify *Tetrahymena* proteins. The peptide mass range was set from 500 Da to 5000 Da. The results were filtered with FDR (false discovery rate) set to 1% at the peptide and protein level. Quantitative and statistical analyses was performed using the IonQuant program and Perseus, respectively (v. 2.0.3) (Max Planck Institute of Biochemistry, Martinsried, Germany). In the label-free method for statistical analysis, proteins with quantitative information present in two out of three samples in at least one group were used. Missing values were filled in based on imputation using the QRILC method. For differential quantitative analysis, a Student’s *t*-test was used. During analysis the fold change cut-off was set at 1 (s0 = 0.5). Whenever the difference between groups had an adjusted *p* value of ≤0.05, proteins were assumed to be differentially expressed.

### 2.9. Quantitative Real-Time PCR and RT-PCR

Total RNA was purified from cells cultured to the mid-log phase, with the Universal RNA Purification Kit (EURX, Gdansk, Poland) and the on-column DNAse digestion protocol provided by the manufacturer. The reverse transcription was carried out with SuperScript III First-Strand Synthesis SuperMix for qRT-PCR kit (Thermo Fisher Scientific Baltics, Vilnius, Lithuania) using 500 ng of purified RNA and oligo dT, according to the manufacturer’s instructions. A real-time RT-PCR was carried out using cDNA as a template and a PowerUp SYBR Green Master Mix (Thermo Fisher Scientific Baltics, Vilnius, Lithuania) with the standard cycling protocol according to the manufacturer’s instruction in StepOnePlus Real-Time PCR System (AB Applied Biosystems, Foster City, CA, USA). Standard curves were prepared with the use of known amounts of housekeeping, GCP4 and analyzed genes DNA (either plasmid or PCR product). The initial amount of cDNA was estimated using Step One Plus Software.

## 3. Results

### 3.1. Identification of Proteins Positioned in Close Proximity to Tetrahymena Cfap91

*Tetrahymena* Cfap91 localizes exclusively in cilia [21]. To identify proteins positioned in close proximity to Cfap91, and thus, to point to likely intraciliary localization of Cfap91, we engineered *Tetrahymena* cells expressing fusion protein, Cfap91-HA-BirA* under the control of a *CFAP91* promoter. The proximity-labeling assay followed by a mass spectrometry-based identification of biotinylated proteins revealed that, in the cilia of Cfap91-HA-BirA* cells, several proteins were efficiently and repeatedly biotinylated (Table 1).

Among biotinylated proteins, the components of the RS3 stalk and the arch-like structure positioned at the RS3 base, Cfap61 and Cfap251, respectively [21], were identified by the highest number of peptides. Additionally, we detected Cfap206, a likely RS2-base component [32] and radial spoke stalk proteins, RSP8 and RSP3 (of note, *Tetrahymena* genome encodes 3 paralogs of RSP3, but here we identified only two of them, RSP3B and RSP3C). Thus, most likely, Cfap91 is located in close proximity to the base of radial spokes RS2 and RS3.

### 3.2. Knockout of CFAP91 Dramatically Reduces Tetrahymena Cell Motility

**Figure 2:**
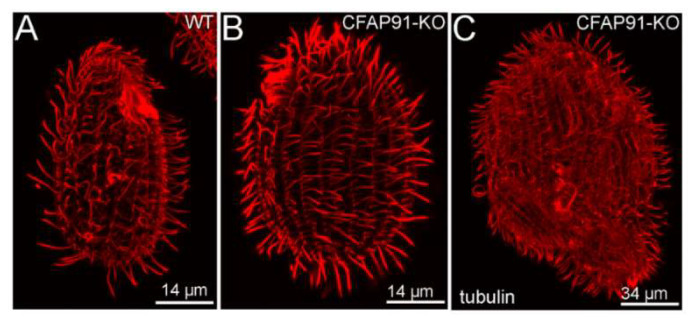
Cytokinesis defects in CFAP91-KO cells population. (**A**–**C**) Immunofluorescence images showing wild-type (**A**) and CFAP91-KO mutant (**B**,**C**) stained with a mix of anti-α-tubulin (12G10) and polyG antibodies to visualize the entire cilia length. (**B**) Note that CFAP91-KO cell (likely reverting from doublet stage) has a larger dimension than wild-type cell (**A**). (**C**) CFAP91-KO monster cell composed of several subcells formed due to defective cytokinesis.

To elucidate the role of Cfap91 in cilia, we knocked out the *CFAP91* gene using a germ-line approach. The phenotypic analysis revealed that *Tetrahymena* CFAP91-KO cells are nearly immotile. Moreover, knockout cells divide very slowly and some of them tend to form doublets (cells were grown in rich MEPP medium [45]) or monster cells composed of several subcells (Figure 2). The inhibition of cytokinesis is a cilia-related defect. At the end of cytokinesis, cilia beating generates a force that enables breakage of the cytoplasmic bridge connecting daughter cells leading to cells separation (so-called rotokinesis [46]). The number of cells that are unable to finish cytokinesis is reduced when cells are grown with moderate shaking (data not shown) which substitutes cilia-generated force supporting cells’ separation.

**Figure 3:**
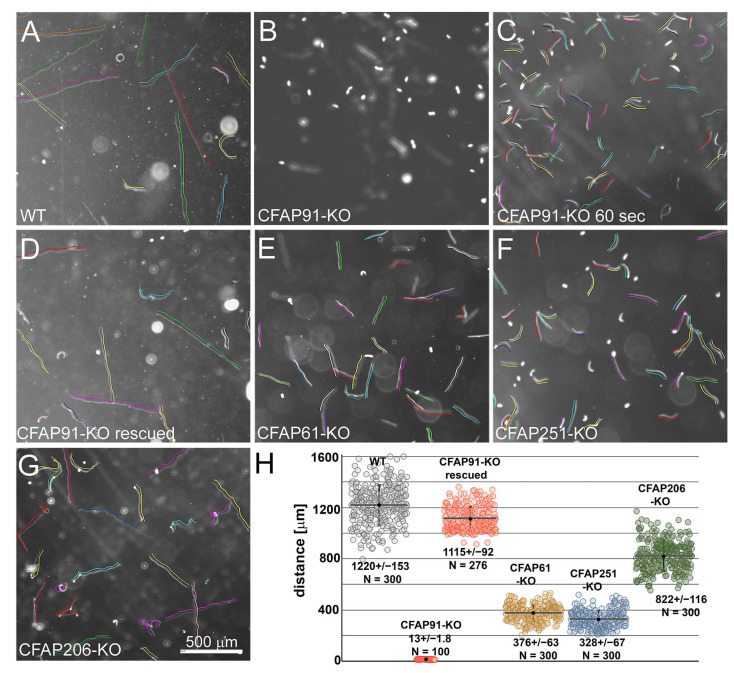
Knockout of genes encoding radial spoke base or radial spoke docking proteins affect *Tetrahymena* swimming to a different extent. (**A**–**G**) Swimming paths of wild-type (**A**) and mutant cells: *CFAP91-KO* (**B**,**C**), *CFAP91-KO* rescued (**D**), *CFAP61-KO* (**E**), *CFAP251-KO* (**F**), and *CFAP206* (**G**). All swimming paths were recorded for 3 s (except for (**C**) where cells were recorded for 60 s) at RT. Cells’ trajectories are indicated by parallel color lines. Bar = 500 μm. (**H**) Graph showing the comparison of the distances traveled by the wild type (WT) and analyzed mutants for 3 s. Error bars represent standard deviation. The traveled distance with standard deviation and number of measured trajectories (N) are indicated. Calculated *p*-value (*t*-test): WT/91-KO 5.67 × 10^−272^, WT/91-res 8.6772 × 10^−264^, WT/61 8.6772 × 10^−264^, WT/251 1.265 × 10^−277^, and WT/206 4.9965 × 10^−147^.

Using dark-field microscopy, we registered the cells’ motility and measured the traveled distance using the ImageJ program. We have found that, while wild-type cells swam on average a distance of 1221.5 ± 155 µm (*n* = 300) for 3 s, at the same time CFAP91-KO cells were nearly immobile (Figure 3A–C,H) and during 60 s covered a distance of only 253.4 ± 34.9 µm (*n* = 100).

Thus, the swimming velocity of CFAP91-KO cells was reduced by almost a hundred times compared to wild-type cells (4.22 µm/s versus 406.9 µm/s). The expression of the transgenic Cfap91-3HA from the non-essential *BTU1* locus [47] restored mutant motility up to 91% (*n* = 276) of the wild-type cells (Figure 3D,H), confirming that the observed phenotype was caused exclusively by a deletion of the fragment of *CFAP91* gene.

The motility of *Tetrahymena* mutants with knocked-out either *CFAP61* or *CFAP251* genes encoding two other subunits of the so-called CSC complex [13,14,21], or *CFAP206* gene encoding a protein essential for RS2 stability [32] was reduced to a lesser extent (Figure 3E–H), which agrees with previous reports [21,32]. In our hands, under the same growing conditions, their swimming velocity was approximately 125 µm/s (CFAP61-KO), 109.3 µm/s (CFAP251-KO), and 274.5 µm/s (CFAP206-KO), that is 31%, 27%, and 67% of the wild-type cell velocity, respectively.

**Figure 4:**
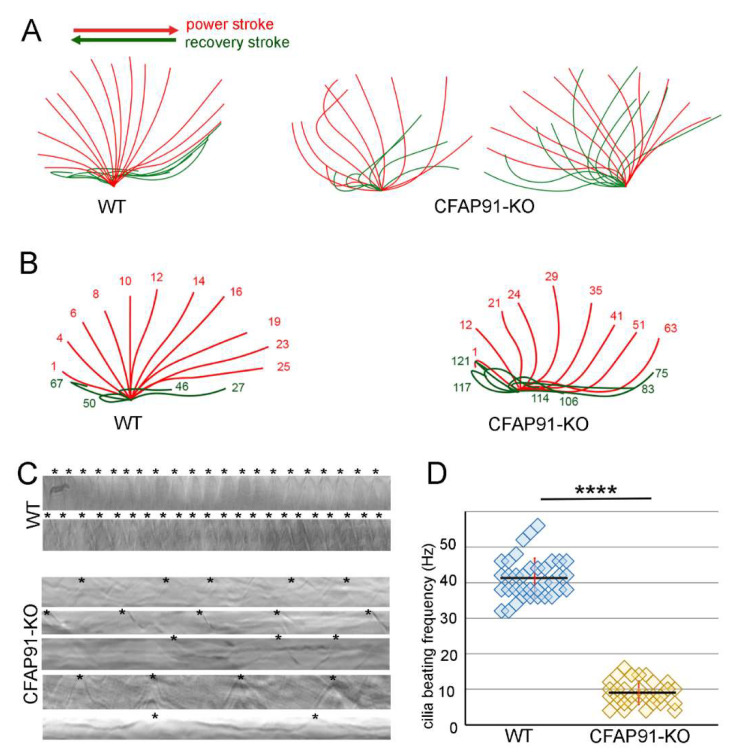
Lack of Cfap91 drastically reduces cilia beat frequency. (**A**) Drawings representing subsequent positions of the cilium during the power (red) and recovery stroke (green) of wild-type and CFAP91-KO mutant cells swimming in SPP medium, prepared based on recorded movies. (**B**) Drawings as in (**A**) but with indicated numbers of movie frames when the particular cilium position was reached. Cells were recorded in a mix of SPP medium and 1.5% methylcellulose. (**C**) Examples of kymographs of cilia motility recorded for 0.5 s (450 frames), analyzed using the ImageJ program, and used to calculate the cilia beating frequency. Ciliary beat cycles marked by stars *. (**D**) Graph showing measurements of cilia beating frequency in wild-type (WT) and CFAP91-KO mutant. Number of analyzed kymographs: WT (5 cells, 36 kymographs), CFAP91-KO (8 cells, 34 kymographs). Calculated *p*-value (*t*-test): WT/91-KO 5.29 × 10^−36^ (indicated by four stars ****).

The detailed analyses of CFAP91-KO mutant ciliary behavior using a high-speed camera showed that cilia beating was significantly altered (Figure 4). In wild-type cells, cilia beat with the metachrony. During the power stroke, a cilium is stiff and its tip marks almost a semicircle (from the anterior to the posterior cell end) in the plane perpendicular to the cell surface. During the recovery stroke, a cilium bends and the position of the bend shifts from the cilium base to the tip as the cilium returns in the plane parallel to the cell surface to its initial position (Figure 4A,B, Supplementary Movie S1).

In the CFAP91-KO mutant, neighboring cilia frequently exhibited different beating behavior. In consequence, cilia tend to lose metachrony and collide affecting both ciliary waveform and frequency. Moreover, some cilia seemed to pause or even slightly move back during the power stroke, struggle to initiate a bend during the recovery stroke, and their beating amplitude was frequently reduced. However, in the case of many cilia, nearly normal beating amplitude could be observed (Figure 4A,B, Supplementary Movie S2).

Other than asynchronous cilia beating, the most striking change caused by the *CFAP91* knockout, was a dramatic reduction of cilia beating frequency. While wild-type cells cilia beat at a frequency of approximately 42 Hz, in mutants (whenever it was possible to measure) the beating frequency ranged from 4 to 16 Hz (Figure 4C,D). Interestingly, a more coordinated cilia beating with a slightly higher beat frequency was observed near the anterior cell end (Figure S2).

The comparative analyses of the cilia beating in *CFAP61*, *CFAP206*, and *CFAP251* knockout cells (Figure S3) clearly indicated that the phenotypic alterations in these mutants are less severe. In the case of CFAP61-KO and CFAP251-KO mutants, the cilia beating frequency was approximately half of this in wild-type cells (20 ± 2.7 and 23 ± 4.6 Hz, respectively) while deletion of *CFAP206* reduced cilia beating by nearly a quarter (32 ± 4.3 Hz).

### 3.3. Cilia of Cfap91 Knockout Mutant Lack Some Radial Spokes and Inner Dynein Arms

**Figure 5:**
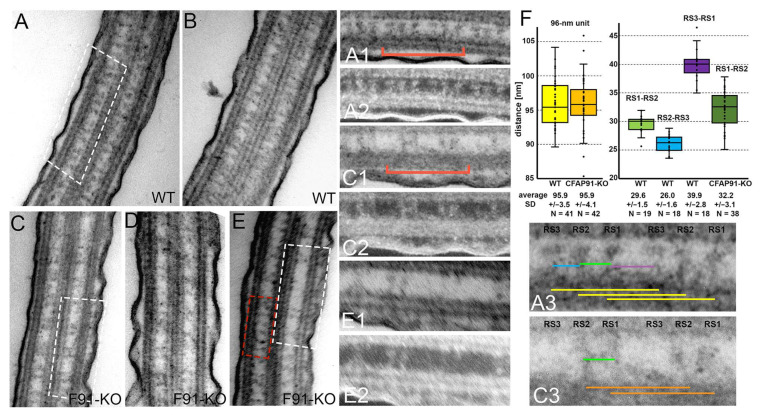
One of the radial spokes is lost in the subset of CFAP91-KO 96-nm ciliary units based on the classical transmission electron microscopy analyses. (**A**–**E**) Longitudinal sections of cilia of wild-type (**A**,**B**) and CFAP91-KO cells (**C**–**E**). A red rectangle box in (**E**) indicates a region where radial spokes are close to each other (no apparent triplets visible). (**A1**,**A2**,**C1**,**C2**,**E1**,**E2**) Magnified fragments of the axonemes are marked by white rectangle boxes in **A**, **C**, and **E**, respectively. (**A2**,**C2**,**E2**) Reverse contrast images of the images presented as (**A1**,**C1**,**E1)**, respectively (**A3**,**C3**) Magnified fragments of the wild-type (**A3**) and mutant (**C3**) axonemes (marked by a red bracket in **A1**,**C1**, respectively) showing two adjacent radial spoke triplets, (**F**) A graph representing measurements of the distances between radial spokes (as indicated by color lines in **A3**,**C3**) in wild-type and CFAP91-KO mutant cilia.

The dramatically altered CFAP91-KO mutant’s swimming behavior is most likely a consequence of the defect(s) in the cilia ultrastructure. The transmission electron microscopy (TEM)-based analysis of cilia longitudinal sections indicated that, in contrast to wild-type axonemes (Figure 5A,B), cilia in CFAP91-KO mutant frequently lack one (likely RS3) and rarely two radial spokes (likely RS2 and RS3) out of the radial spoke triplet (Figure 5C–E). However, in some longitudinal sections, radial spokes seemed to be docked more densely on the outer doublet (Figure 5E, red frame). Based on the fact that the ciliary unit (here measured as a distance between RS1-RS1, RS2-RS2, and RS3-RS3 of two adjacent radial spoke triplets (Figure 5A3,C3, yellow and orange lines) has a length of 96 nm, we estimated a distance between particular radial spokes in the cilia of both, wild-type and CFAP91-KO mutants (Figure 5F). In wild-type cells, RS1 was 29.6 ± 1.5 nm apart from RS2, RS2 was 26 ± 1.6 nm from RS3 and a gap between RS3 of one triplet and RS1 of the adjacent triplet was 39.9 ± 2.8 nm (these values are in good agreement with previously reported distances between radial spokes: 32 nm (RS1-RS2), 24 nm (RS2-RS3), and 40 nm (RS3-RS1) [48]. In CFAP91-KO cells, the two existing radial spokes were positioned 32.2 ± 3.1 nm apart suggesting that these spokes are most likely, RS1 and RS2, although the distance between them was slightly larger than in wild-type cells (*p*-value = 0.00015).

To investigate if indeed RS3, and in some triplets, also RS2, are missing in CFAP91-KO cilia, we analyzed the levels of RS2 and RS3 radial spoke proteins, Cfap61, Cfap206, and Cfap251 using mass spectrometry (the localization of Cfap61, Cfap206, and Cfap251 was recently established using the cryo-ET approach [21,32]. First, we verified that the levels of proteins building structures that are unlikely to be affected in CFAP91-KO cilia such as central apparatus or tether/tether head (T/TH) complex, are similar in control and mutant ciliomes (Table 2). The ratio of detected peptides (CFAP91-KO/WT) oscillated between 0.83–1.1, and thus, the obtained data are comparable.

As expected, the Cfap91 protein was missing in CFAP91-KO mutant cilia (Table 2). Moreover, Cfap61, known to build part of the RS3 stalk, and Cfap251, a component of the arch-like structure positioned at the RS3 base [21], were significantly reduced in CFAP91-KO mutant cilia. Interestingly, the level of Cfap206, a protein required for the stability of RS2 and partly RS3 [32] was also diminished in CFAP91-KO mutant cilia. These proteomic data nicely match our TEM analyses suggesting that RS3 or RS3 and RS2 are missing in cilia of CFAP91-KO mutant and thus Cfap91 likely functions as an RS3 adaptor protein.

**Figure 6:**
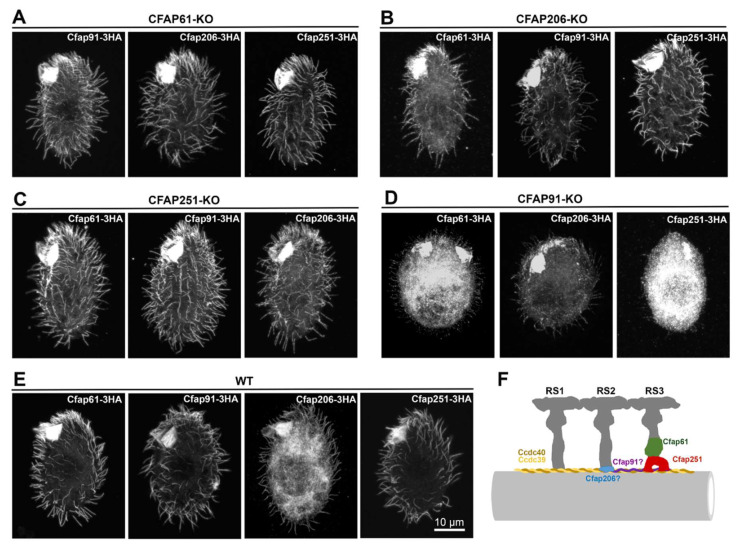
Ciliary localization of Cfap61, Cfap206, and Cfap251 is strongly reduced in cells lacking Cfap91. Immunofluorescence confocal images showing localization of the HA-tagged fusion proteins expressed under the control of respective native promoters in cells with deletion of (**A**) *CFAP61*, (**B**) *CFAP206*, (**C**) *CFAP251*, or (**D**) *CFAP91,* and in (**E**) wild-type cells. Note that only the presence of Cfap91 is required for the ciliary localization of other studied Cfap proteins. (**F**) Schematic representation of the outer doublet microtubule (light grey) with three radial spokes (dark grey) showing confirmed (Cfap61, Cfap251) and putative (Cfap206 and Cfap91) localization of studied proteins. The proposed model explains why Cfap91 could be a key protein required for Cfap61, Cfap206, and Cfap251 stable docking. Ccdc39 and Ccdc40 (yellow and brown, respectively) are molecular ruler proteins, determining radial spokes position [49], Cfap206 (blue) is required for the stable assembly of RS2 [32], Cfap61 (green) and Cfap251 (red) form part of the RS3 stalk and arch-like structure at RS3 base, respectively [21], Cfap91 (purple) interacts with Ccdc39/Ccdc40 [12] and play a role in the stable assembly of RS2 and RS3-base proteins.

Next, to further verify ultrastructural and mass spectrometry data, we expressed all four studied proteins in their native loci (to ensure the expression under the control of native promoter) as C-terminal 3HA fusions in wild-type cells and *CFAP61*, *CFAP91*, *CFAP206*, and *CFAP251* knockout backgrounds (Figure 6). The immunolocalization studies of 3HA fusion proteins showed that Cfap61, 91, 206, and 251 proteins were present in CFAP61-KO, CFAP206-KO, and CFAP251-KO mutant cilia as in the cilia of wild-type cells, but were hardly detectable in CFAP91-KO mutant cilia, which agrees with proteomic data. Thus, likely, in *Tetrahymena* cilia, Cfap91 is positioned at the base of RS3 and perhaps also RS2 and is crucial for the stable docking of RS3 and, to a lesser extent, RS2 to the outer doublet microtubules (Figure 6F). Moreover, because, with the exception of CFAP91-KO cells, studied proteins are targeted to cilia in CFAP61-KO, CFAP206-KO and CFAP251-KO mutants, most likely all four proteins are transported to cilia independently, and Cfap91 is necessary for the stability of the Cfap61, Cfap206, and Cfap251-containing ciliary structures. These data also suggest that Cfap91 binds to the axoneme before Cfap61, Cfap206, and Cfap251.

Previous cryo-ET and proteomic analyses of *Tetrahymena* mutants revealed that IDA d and IDA g densities were weaker in the averaged 96-nm repeats of CFAP61-KO mutant cilia [21], while IDA c density was missing in the ciliary units of CFAP206-KO cells [32]. The structural defects in CFAP206-KO cilia coincided with the lack of Dyh10, Dyh12 and Dyh25 dynein heavy chains [32].

Our proteomic analysis showed that, in CFAP91-KO mutant cilia, the levels of several dynein heavy chains predicted to form single-headed IDAs, were either significantly or moderately reduced. These were: Dyh10, 12, 13, 16, 22, 23, 24, and 25, and to a lesser extent, Dyh8, Dyh11, and Dyh20 (Table 2). In *Chlamydomonas*, dynein docked near RS3 (dynein d and dynein g) are composed of DHC2 and DHC7, respectively [50]. The bioinformatics studies showed that *Chlamydomonas* DHC7 groups with *Tetrahymena* Dyh22, 23, and 24, while DHC2 with *Tetrahymena* Dyh16 [51]. Thus, at least IDA c, IDA d, and IDA g are missing in most of the CFAP91-KO 96 nm ciliary repeats. Of note, the assignment of Dyh10 to dynein c must be done with caution due to its low abundance even in the wild-type extract. The absence of some types of IDAs in CFAP91-KO cilia are further supported by the fact that the level of the p28B protein, a component of the single-headed inner dynein arms [52] is also reduced. For comparison, the levels of dynein heavy chains, the components of the outer dynein arms (ODAs), Dyh3, Dyh4, and Dyh5, and heterodimeric inner dynein arm, IDA f/I1, Dyh6 and Dyh7 were as in wild-type cells, suggesting that both ODAs and IDA f/I1 were unaffected in CFAP91-KO mutant.

To sum up, the mass spectrometry and TEM data indicate that alteration in the ciliary beating, and as a consequence, almost a complete paralysis of CFAP91-KO cells, are caused by the major ciliary ultrastructural defects. Those include: (i) frequent lack of RS3 and rarely, RS2, the ciliary complexes participating in the transduction of mechano-chemical signals from the central apparatus to dynein arms, and (ii) reduced number of some of single-headed IDAs, the effector structures. We cannot exclude other structural defects in the RS2-RS3 vicinity in CFAP91-KO mutant cilia.

Interestingly, the phenotypic outcomes of the deletion of *CFAP61*, *CFAP91,* and *CFAP251* genes are strikingly different. Moreover, in *Tetrahymena*, besides Cfap61 and Cfap251, ciliary localization of Cfap206 also depends upon the presence of Cfap91 protein. This raises the question of whether Cfap61, Cfap91, and Cfap251 do indeed function as a separate CSC complex or are simply part of the radial spoke structure (Cfap61, Cfap251, as proposed by [18]) or positioned in the RS vicinity (Cfap91, RSs adaptor protein).

### 3.4. Regulation of Cfap61, Cfap91, and Cfap251 Proteins in Tetrahymena

The Northern blot-based studies of the levels of *FAP61* and *FAP91* transcripts in the wild-type and *Chlamydomonas* amiRNA *fap61* (CaM-IP3) and *fap91* (CaM-IP2) knockdowns suggested co-regulation of the transcription of both genes [14]. To investigate if a similar mode of regulation exists in *Tetrahymena*, we purified RNA from wild-type and knockout mutants and performed qRT-PCRs to estimate the levels of expression of *CFAP61*, *CFAP91*, and *CFAP251*.

**Figure 7:**
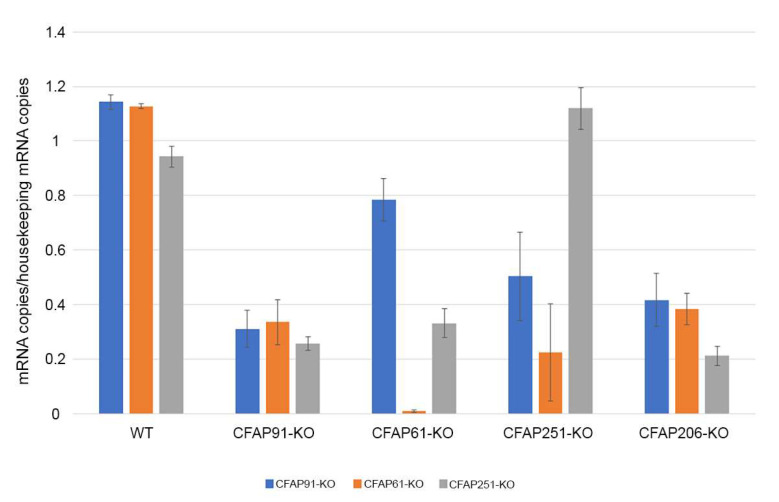
Real-time RT-PCR analyses of the levels of expression of *CFAP61*, *CFAP91*, and *CFAP251*. *GCP4* was used as a housekeeping gene.

The quantification of the levels of *CFAP61*, *CFAP91*, and *CFAP251* mRNAs (cDNA) in the wild-type and knockout cells revealed that, with two exceptions, the levels of mRNAs were significantly reduced in studied mutants, including *CFAP206-KO* (Figure 7). Interestingly, the level of *CFAP91* mRNA was only moderately reduced in CFAP61-KO, while the level of *CFAP251* mRNA was slightly elevated in CFAP251-KO, indicating that the produced aberrant mRNA is stable. However, based on proteomic studies (Table 2), the aberrant or truncated Cfap251 protein if synthesized is not transported to cilia but is likely degraded. Thus, most likely in *Tetrahymena*, as in *Chlamydomonas*, the expression of these genes is co-regulated.

**Figure 8:**
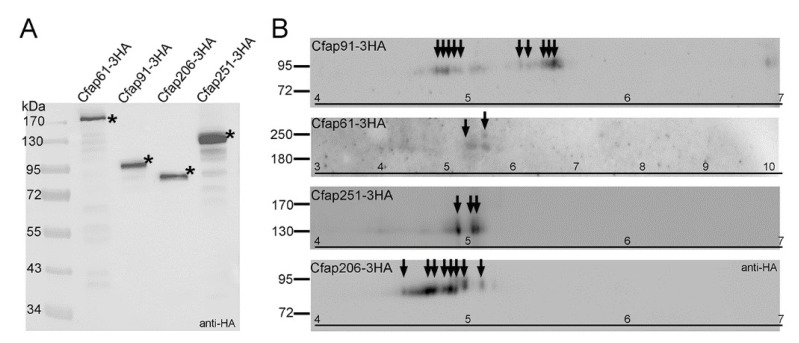
Proteins building the base of RS2 and RS3 are present in cilia in more than one isoform. (**A**) The Western blot analyses of ciliary proteins isolated from cells expressing C-terminally 3HA tagged Cfap61, Cfap91, Cfap206, or Cfap251 under the control of the respective native promoters. Stars indicate the position of the band of expected size. Weakly visible bands of lower molecular weight are products of protein degradation. (**B**) Two-dimensional analyses of axonemal proteins purified from cells expressing -3HA fusion proteins under the control of the respective native promoters. Isoelectric focusing was performed using 7 cm 4–7 or 3–10 ready-strips. The theoretical pI and Mw were calculated using https://web.expasy.org/compute_pi (accessed on 10 October 2022) (Table 3). Black arrows indicate detected isoforms. Whole blots are presented in Figure S4.

Protein posttranslational modifications (PTMs) change protein properties and/or activity. Cfap91 and Cfap206 are likely radial spoke adaptor proteins (this study and [32]), while Cfap61 and Cfap251 are structural components of RS3 that might play a role in the transduction of the regulatory signals. Therefore, to shed some light on the modes of the Cfaps 61, 91, 206, and 251 regulation, we investigated if those proteins are posttranslationally modified in cilia. We collected cilia from wild-type cells expressing C-terminally 3HA-tagged proteins under the control of the respective native promoter (Figure 6E). The 2-dimensional electrophoresis of the ciliary proteins followed by a Western blot and anti-HA antibody-based detection revealed that, in *Tetrahymena* cilia, all studied Cfap proteins exist in several isoforms (Figure 8), suggesting that they are posttranslationally modified.

Based on the number of detected spots, it seems that Cfap91-3HA and Cfap206-3HA are highly posttranslationally modified, while in the case of Cfap251-3HA, only three isoforms were detected. The largest protein, Cfap61-3HA was prone to degradation, but even in this case we have detected at least two protein isoforms. Thus, proteins that might function as radial spokes adaptors are extensively modified, while in the case of RS3 structural proteins, modifications seem to be scarce. The identification of Cfap91 and Cfap206 modifications and analyses of their significance may help to more fully elucidate the mechanism(s) governing radial spokes positioning and docking to the axoneme.

### 3.5. Cfap91 Interacts Directly with Molecular Ruler Protein, Ccdc39

**Figure 9:**
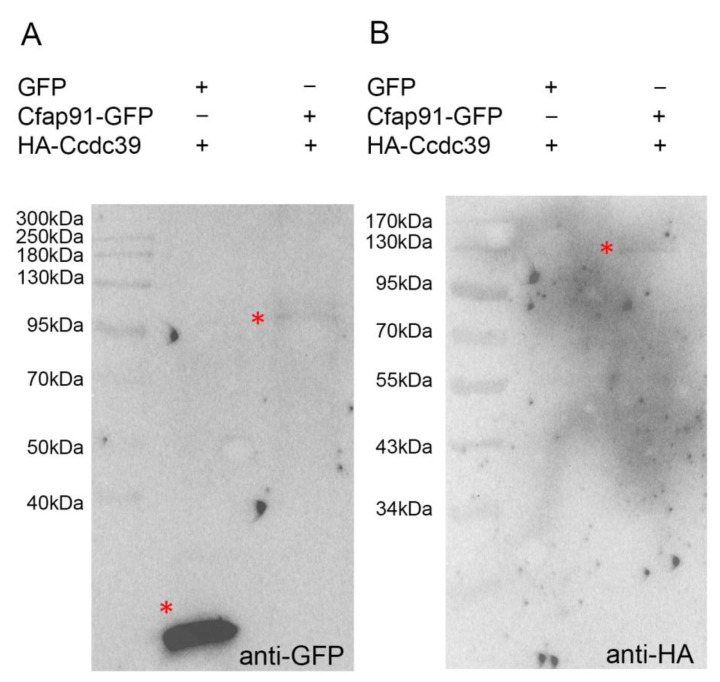
Cfap91-GFP interacts with HA-tagged molecular ruler protein, Ccdc39. GFP and Cfap91-GFP proteins were purified from the cytoplasm of GFP or Cfap91-GFP overexpressing cells using magnetic agarose beads-conjugated anti-GFP antibodies and after washings, incubated with a cytoplasmic extract obtained from cells overexpressing HA-Ccdc39 proteins (118 kDa). (**A**) A Western blot showing that either GFP (26 kDa, first line) or Cfap91-GFP (102 kDa, second line) proteins (both indicated by a red star) were bound to the magnetic beads. (**B**) A corresponding Western blot showing that HA-Ccdc39 was pulled-down by the GFP-Cfap91 protein but not by GFP alone.

Based on the molecular modeling, it was proposed that, in *Chlamydomonas,* FAP91 interacts with molecular ruler proteins, FAP59/CCDC39 and FAP172/CCDC40 [12]. To investigate if Cfap91 and a molecular ruler component, Ccdc39, do indeed interact with each other, we purified GFP, Cfap91-GFP and HA-Ccdc39 proteins from the cytoplasmic fraction of the overexpressing cells, and performed pull-down experiments. A GFP and Cfap91-GFP proteins bound to the GFP-Trap magnetic agarose served as baits to pull down the HA-tagged Ccdc39 protein from the cytoplasmic fraction of HA-Ccdc39 overexpressing cells. The Western blot analyses revealed that Cfap91-GFP but not GFP alone pulled down ~118 kDa HA-Ccdc39 protein (Figure 9). Thus, *Tetrahymena* Cfap91 interacts with a molecular ruler protein, Ccdc39.

*Chlamydomonas* FAP61, FAP91, and FAP251 were co-immunoprecipitated from *pf14* mutant high-salt flagellar extract using anti-CaM antibodies [13], suggesting that one or more of these proteins bind(s) calmodulin. In *Tetrahymena*, calmodulin CaM1 (TTHERM_00630500) is present in cilia (supplementary Tables S2 and S3 [53]. In silico analyses of the *Tetrahymena* orthologs using the Calmodulin Target Database (http://calcium.uhnres.utoronto.ca/ctdb/ctdb/sequence.html (accessed on 15 September 2022)) predicted the presence of potential calmodulin binding sites in all three proteins: Cfap61 F287-D300 (FLNNGKAIVGQVRD), Cfap91 E405-I422 (EAVLLLQRLLRGRTTQNI), and Cfap251 K689-P706 (KYLAYSTKEKVVGIIKLP). However, we were unable to pull down calmodulin from the cytoplasmic fraction obtained from cells overexpressing HA-CaM1 using GFP-tagged Cfap61, Cfap91, or Cfap251 bound to the GFP-Trap magnetic agarose beads (data not shown).

## 4. Discussion

CFAP91/FAP91/MAATS1 protein is a highly evolutionarily-conserved component of motile cilia and flagella. However, there are striking differences in the predicted amino acid composition of orthologous proteins from organisms belonging to diverse evolutionary lineages (Table S1). In the analyzed species belonging to Metamonada, Oomycota, and Ciliophora, the N-terminal fragment is about 60–100 amino acids shorter, and in the case of ciliates, lacks a highly conserved motif RXXD A/Y L/V YDP. This motif is also missing in Trypanosomas FAP91 orthologs. On the other hand, in species belonging to Viridiplantae, including *Chlamydomonas*, FAP91 orthologs are larger and have an unusual amino acid composition in their middle and C-terminal parts. In *Chlamydomonas,* such a striking difference in FAP91 amino acid composition coincides with an unusual RS3 structure resembling a short knob, and thus, with a presence of only two spokes, RS1 and RS2, that can interact with and transduce signals from the central apparatus. To our knowledge, it is yet unknown if in other species belonging to Viridiplantae whose genome encodes *Chlamydomonas*-like FAP91, the RS3 structure is also reduced. Thus, the correlation between FAP91 amino acid composition and assembly of the RS3 in reduced form remains an open question.

CFAP91 orthologs are indispensable for the cilia/flagella motility and cell movement. *Tetrahymena CFAP91* knock-out cells are hardly motile similar to tetracycline-induced *T.brucei TbCFAP91* knock-down mutant [19]. In contrast, *Chlamydomonas* amiRNA knock-downs can swim slowly and the forward swimming velocity varies between mutant strains [14]. The abnormal cell movement is a consequence of changes in cilia/flagella beating, although these changes are not the same in the studied species. In *Tetrahymena* CFAP91-KO mutant ciliary frequency is strongly reduced, while in the *Chlamydomonas* mutant flagella, the beating frequency is only slightly reduced. However, in both these species, deletion/depletion of FAP91 causes cilia/flagella asynchrony, and alters cilia movement during the power and recovery strokes [14].

The ultrastructural defects (TEM) also vary in the analyzed *FAP91* mutants. In *Trypanosoma*, knock-down of *TbCFAP91* causes rotation of the central apparatus. Regrettably, the number and position of radial spokes in this mutant were not analyzed [19]. Interestingly, rotation of the central apparatus was not reported in *T.brucei RSP3* knock-down mutant [54] raising a question about the mechanisms behind the central apparatus rotation in the *TbCFAP91* mutant.

In humans, truncation of CFAP91 leads to MMAF due to defects in sperm cells, while PCD symptoms were not reported. In affected individuals, sperm flagella axonemes were frequently disorganized and some of the analyzed flagella cross-sections lacked the central apparatus [19]. The organization of the radial spokes in sperm cell flagella was not addressed at the ultrastructural level, but using the immunofluorescence approach it was shown that RSPH1 and CFAP251 radial spoke proteins were either absent or significantly reduced [19], suggesting either defects or lack of radial spokes.

The ultrastructural analysis of the *Tetrahymena* CFAP91-KO cells using classic TEM revealed that one of the radial spokes, RS3, is missing in numerous 96-nm axonemal units. These data are supported by the comparative total ciliome proteomic studies showing that the levels of Cfap61, the RS3 stalk protein, and Cfap251, the component of the arch-like structure at the RS3 base [21] are significantly reduced in the CFAP91-KO cilia. Interestingly, the level of Cfap206 protein is also significantly reduced in CFAP91-KO mutants. It was previously reported that RS2 is missing in most of the *Tetrahymena* CFAP206-KO axonemal units [32]. These data agree with our observations that occasionally only one radial spoke was visible in CFAP91-KO cilia longitudinal sections, suggesting the lack not only of RS3 but also of RS2 (or RS2 base and a stalk part) in mutant cilia. These data largely agree with cryo-electron microscopy analyses of the flagella from *Chlamydomonas* mutants with knocked-down *FAP91.* Although the most striking ultrastructural aberration in *Chlamydomonas* amiRNA *fap91* mutant was lack of RS2, the remnant structure of knob-like RS3 was also missing or reduced [14,18]. Finally, rarely, in both *Chlamydomonas* [18] and *Tetrahymena* (this study), radial spokes seem to be docked in an irregular way [14,18].

Based on the molecular modeling, it was proposed that *Chlamydomonas* FAP91 forms a triple helix with outer doublet molecular ruler proteins, CCDC39/FAP59 and CCDC40/FAP172, and likely docks RS2 to the axoneme [12]. Our pull-down experiments confirm that *Tetrahymena* Cfap91 can interact with Ccdc39 supporting this model.

Taken together we suggest that in *Tetrahymena* Cfap91 protein interacts with the molecular ruler and mediates docking of RS3, and to a lesser extent, of RS2. As a consequence, its deletion causes either a lack of radial spokes or their irregular docking. Interestingly, we found that the distance between RS1 and RS2 estimated based on TEM images is slightly larger in CFAP91-KO mutants than in wild-type cells, suggesting the possibility of the shift of RS2 with respect to RS1. These findings further support the role of Cfap91 in RS2 docking.

The comparative proteomic analyses of dynein heavy chains revealed that the levels of several dynein heavy chains, components of single-headed IDAs, were reduced (in contrast to these in ODAs and heterodimeric IDA f/I1). Thus, lack of the Cfap91 affected not only the stable attachment of radial spokes but also specific IDAs docked at the radial spoke base: IDA d and g docked at RS3 and IDA c docked at RS2. We can’t exclude that other single-headed IDAs are also affected as the level of several unassigned dynein heavy chains was also reduced in CFAP91-KO mutant. To summarize, based on the ultrastructural and proteomic data it seems that lack of Cfap91 protein affects stable docking of outer doublet complexes, two radial spokes and adjacent IDAs and thus interrupts the transduction of regulatory signals from the central apparatus to the dynein arms.

In *Chlamydomonas,* FAP91 was originally described as a protein that can be co-immunoprecipitated with FAP61 and FAP251 from *pf14* spokeless mutant high-salt flagellar extract using anti-CaM antibodies [13]. When wild-type flagellar extract was used, *Chlamydomonas* FAP91 co-immunoprecipitated also with radial spoke proteins. Based on these data, it was proposed that all three proteins form so-called calmodulin and spoke-associated complex (CSC). Intriguingly, the anti-CaM antibody (but not anti-FAP91/CAM-IP2 antibody) precipitated significantly less of FAP61, FAP91, and FAP251 proteins from flagella of *pf2* or *pf3* N-DRC mutants [13]. The cryo-ET analyses of *pf2* and *pf3* mutant axonemes did not reveal any defects in the mutant radial spoke ultrastructure ([55], Figure 4, compare 4P (wild type) with 4S (*pf2*) and 4T (*pf3*)). This raises the question as to why, when proteins were purified from *pf2* and *pf3* flagella lacking most of the N-DRC structure and some IDAs (dynein e/IA4 and dynein g/IA5), the anti-CaM antibodies precipitated less of CSC subunits, although their level in those flagella was as in the wild-type flagella [13].

In contrast to the overlay assay that proved a direct binding between FAP91/CAM-IP2 and RSP3 (both proteins were expressed in bacteria), the interaction between *Chlamydomonas* CaM and FAP91 were studied using flagellar extracts. Thus, one cannot exclude the possibility that calmodulin binds to N-DRC, which in turn connects with one or more CSC components. This would explain a poor recovery of CSC subunits from N-DRC mutant (*pf2* or *pf3*) axonemes using anti-CAM antibodies despite no alterations in FAP61, FAP91, and FAP251 levels.

Interestingly, the calmodulin was not identified among ciliary proteins biotinylated in *Tetrahymena* cells expressing Cfap91-HA-BirA*. Moreover, we could not show interactions between calmodulin and CSC subunits using a pull-down assay and GFP or HA-tagged proteins purified from the cytoplasm of overexpressing cells. However, although CaM1 is a ciliary protein, we cannot exclude that Cfap91, Cfap61, and/or Cfap251 could interact with calmodulin-like proteins and that the levels of calmodulin-like proteins were too low to be detected in our BioID experiment. Another possibility is that the N-terminal HA tagging of calmodulin affected protein–protein interactions.

To sum up, we propose that Cfap91 interacts with different ciliary structures (96-nm molecular ruler, RS2 and RS3 bases) and is indispensable for the stable attachment of the RS3 and to a lesser extent RS2. Defects in radial spokes, in turn, weaken the attachment of the radial spoke associated IDAs. Thus, lack of Cfap91 causes major ultrastructural aberrations of the axoneme leading to abnormal cilia beating behavior.

## Figures and Tables

**Table 1:** Mass spectrometry-based identification of ciliary proteins biotinylated in cells expressing the Cfap91-HA-BirA* fusion protein.

Protein Name	Localization	Number in TGD	Mw (kDa)	Number of Identified Peptides					
				WT			Cfap91-HA-BirA*		
				Exp 1	Exp 2	Exp 3	Exp 1	Exp 2	Exp 3
Cfap91	RS base	TTHERM_00578560	76	0/0	0/0	0/0	11/10	7/6	11/10
Cfap206	RS2 base	TTHERM_00820660	74	0/0	0/0	0/0	9/9	5/5	12/12
Cfap61	RS3 stalk	TTHERM_00641200	199	0/0	0/0	0/0	26/24	13/13	22/22
Cfap251	RS3 base	TTHERM_01262850	115	0/0	0/0	0/0	22/22	12/12	12/11
Rsp3B	RS stalk	TTHERM_00566810	80	0/0	0/0	0/0	7/7	4/4	13/11
Rsp3C	RS stalk	TTHERM_00418270	111	0/0	0/0	0/0	15/15	9/9	9/7
Rsp8	RS stalk	TTHERM_00313520	65	0/0	0/0	0/0	6/6	4/4	8/7
EF-hand	unknown	TTHERM_00492840	67	0/0	0/0	0/0	9/9	5/3	7/7
Hcfc1-like	unknown	TTHERM_00760390	48	0/0	0/0	0/0	4/4	2/2	3/3
Ak8	unknown	TTHERM_00227800	50	0/0	0/0	0/0	20/18	11/11	1/1
Gk1	unknown	TTHERM_00781030	31	0/0	0/0	0/0	6/5	2/2	3/3
𝛼-tubulin	MT	TTHERM_00558620	50	4/4	21/11	3/3	14/13	10/9	5/5
β-tubulin	MT	TTHERM_00348510	50	4/4	21/9	4/4	16/14	11/11	8/7

Table shows only proteins that were biotinylated in all three experiments. MT—microtubule, RS—radial spoke. Numbers X/Y: (X) number of all identified peptides (in a Mascot program, all significant matches), (Y) number of all unique peptide sequences (in a Mascot program, significant sequences). TTHERM—accession numbers in Tetrahymena Genome Database (TGD).

**Table 2:** The levels of proteins associated with radial spokes base and dynein arms in wild-type and CFAP91-KO mutant cilia.

Name.	TGD IDTTHERM_	Subunit of the Structure	Number of Peptides		Ratio KO/WT	Mean Number of Peptides from 3 Replicates		Ratio KO/WT
			Exp. 1			Exp. 2		
			WT	91-KO		WT	91-KO	
Cfap91	00578560	RS2/RS3 base	33	0	**0**	32	0	**0**
Cfap206	00820660	RS2 base	31	1	**0.032**	27.7	0.3	**0.01**
Cfap61	00641200	RS3 stalk	61	10	**0.164**	60	8.7	**0.14**
Cfap251	01262850	arch at the RS3 base	51	3	**0.059**	47.7	0.3	**0.007**
**Outer Dynein Arms (ODA) Heavy Chains**								
Dyh3	01276420	ODA	284	258	0.908	314	263	0.86
Dyh4	00499300	ODA	302	272	0.9	335	290	0.86
Dyh5	00486600	ODA	252	244	0.968	278	221	0.79
**Two-Headed Inner Dynein Arm (IDAf/I1) Heavy Chains**								
Dyh6	00688470	IDA f/I1	170	145	0.853	158	157	0.99
Dyh7	00912290	IDA f/I1	166	136	0.819	148	152	1.02
**Single-Headed Inner Dynein Arms (IDA) Heavy Chains**								
Dyh8	00531870	?	13	8	**0.615**	14.3	8.7	**0.6**
Dyh9	00947430	?	29	28	0.966	23.7	37.3	**1.58**
Dyh10	00420340	dynein c	8	1	**0.125**	12	3	**0.25**
Dyh11	00252430	?	121	83	**0.686**	124	89	0.72
Dyh12	00919540	dynein c	56	4	**0.071**	46.7	3	**0.06**
Dyh13	01104900	?	0	0	-	12	2.7	**0.22**
Dyh14	00492830	?	53	58	1.094	38.7	49	**1.26**
Dyh15	00433800	dynein g	150	129	0.86	152	110	0.72
Dyh16	00558640	dynein d	122	49	**0.402**	93.7	45	**0.48**
Dyh17	00850620	?	0	0	-	0	0	-
Dyh18	00047540	?	3	3	1	0	0	-
Dyh19	01027670	?	112	81	0.723	92.3	91.7	0.99
Dyh20	00821980	dynein d	23	14	**0.609**	19	16	0.84
Dyh22	00565600	dynein g	130	44	**0.338**	113	55	**0.49**
Dyh23	00355100	dynein g	5	2	**0.4**	7.3	3.7	**0.5**
Dyh24	00193520	dynein g	95	27	**0.284**	85.7	22.7	**0.26**
Dyh25	00774810	dynein c	61	4	**0.066**	58.3	10.3	**0.18**
p28A	00841210	IDA	19	9	**0.47**	17	14.3	0.84
p28B	00319990	IDA	8	3	**0.38**	17.3	6.3	**0.37**
p28C	01129720	IDA	14	11	0.78	15.7	8.7	**0.55**
**Central Pair (CP) and Tether/Tether Head (T/TH) Proteins**								
Pf16	000157929	CP	24	20	0.833	37.7	32.7	0.87
Pf20	00134890	CP	33	35	1.060	29.3	23.7	0.81
Hydin	00551040	CP	183	202	1.103	159.7	169.3	1.06
Cfap43	00196190	T/TH	80	70	0.87	65.3	69.7	1.07
Cfap44	00498220	T/TH	90	81	0.9	82	72.7	0.88

Color code: the level of proteins in CFAP91-KO cilia is: (i) red: one fourth or less of the level in the wild-type cilia; (ii) green: 25–50% of the level in the wild-type cilia; (iii) blue: 51–70% of the level in the wild-type cilia; (iv) purple: elevated in CFAP91-KO cilia compared to wild-type cilia.

**Table 3:** Calculated theoretical pI and Mw of analyzed proteins.

Calculated pI/Mw:	Cfap91	Cfap61	Cfap251	Cfap206
without a 3HA tag	7.25/76 kDa	5.41/198 kDa	4.75/114 kDa	6.03/73 kDa
with a 3HA tag	6.23/79.5 kDa	5.32/202 kDa	4.68/118 kDa	5.48/77 kDa

## Data Availability

All data and *Tetrahymena* strains are available on request.

## Supplementary Materials

The following supporting information can be downloaded at: https://www.mdpi.com/article/10.3390/cells11244048/s1, Figure S1: Multiple alignment of Cfap91 homologs (alignment was prepared using ClustalX2 [56] and SeaView [57] and amino acid residues were shaded using GeneDoc [58]; Figure S2: Analyses of cilia beating in CFAP91-KO cells; Figure S3: Lack of Cfap61, Cfap206 or Cfap251 proteins reduces cilia beat frequency; Figure S4: Whole blots used in Figure 8B; Table S1: Primers used to amplified genes coding region; Movie S1: Cilia beating in wild-type cell. Playback speed 25 fps; Movie S2: Cilia beating in CFAP91-KO cell. Playback speed 25 fps.
