# Supplementary material for: Cfap91-Dependent Stability of the RS2 and RS3 Base Proteins and Adjacent Inner Dynein Arms in Tetrahymena Cilia
Source: Cells. 2022 Dec 14;11(24):4048. doi: 10.3390/cells11244048 (PMC9776847; doi:10.3390/cells11244048)
Supplement: Supplementary file 1 [file cells-11-04048-s001.zip › cells-2019672 Supplementary Materials 2.pdf]

Supplementary Data

Figure S1. Multiple alignment of Cfap91 orthologs.

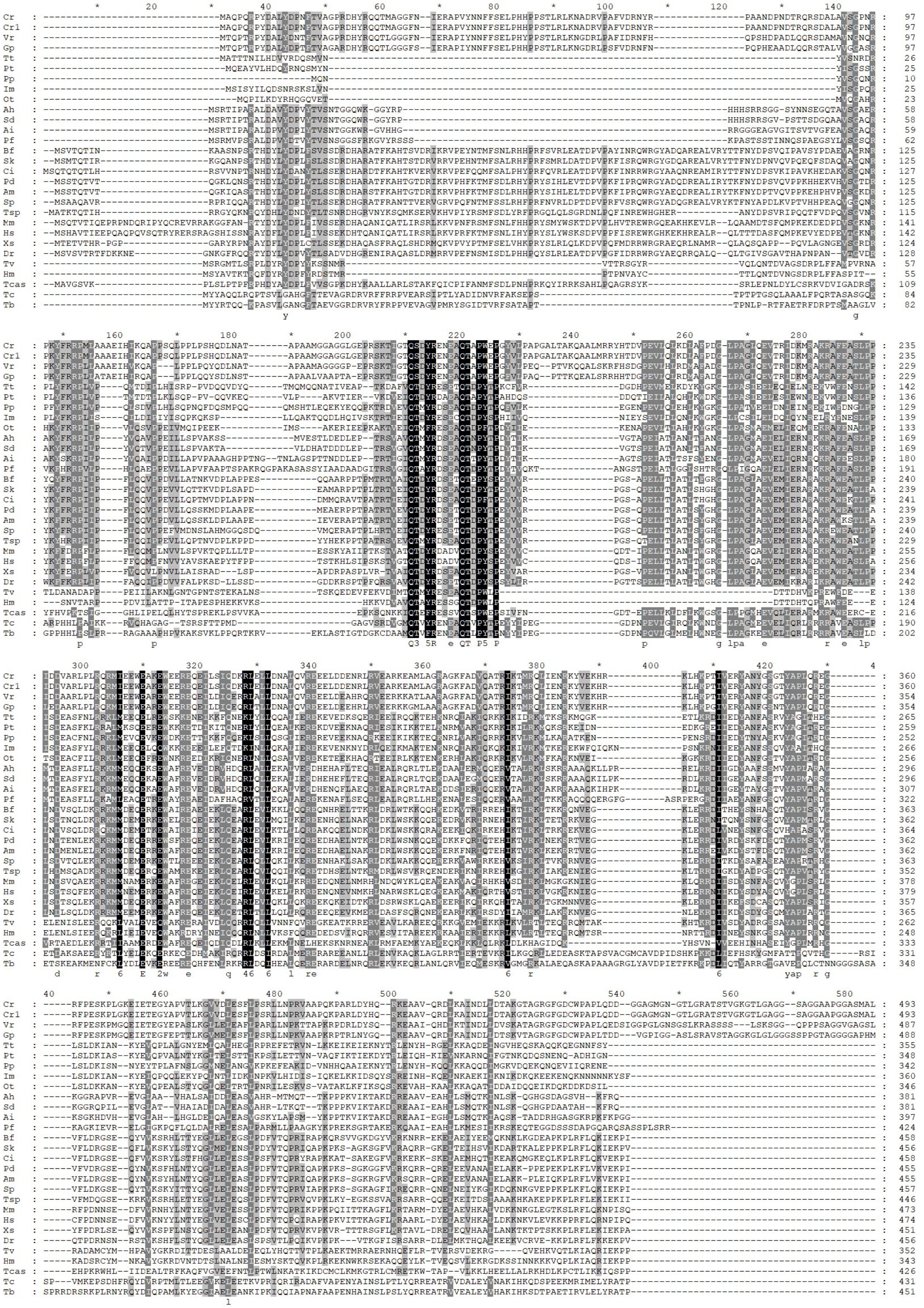

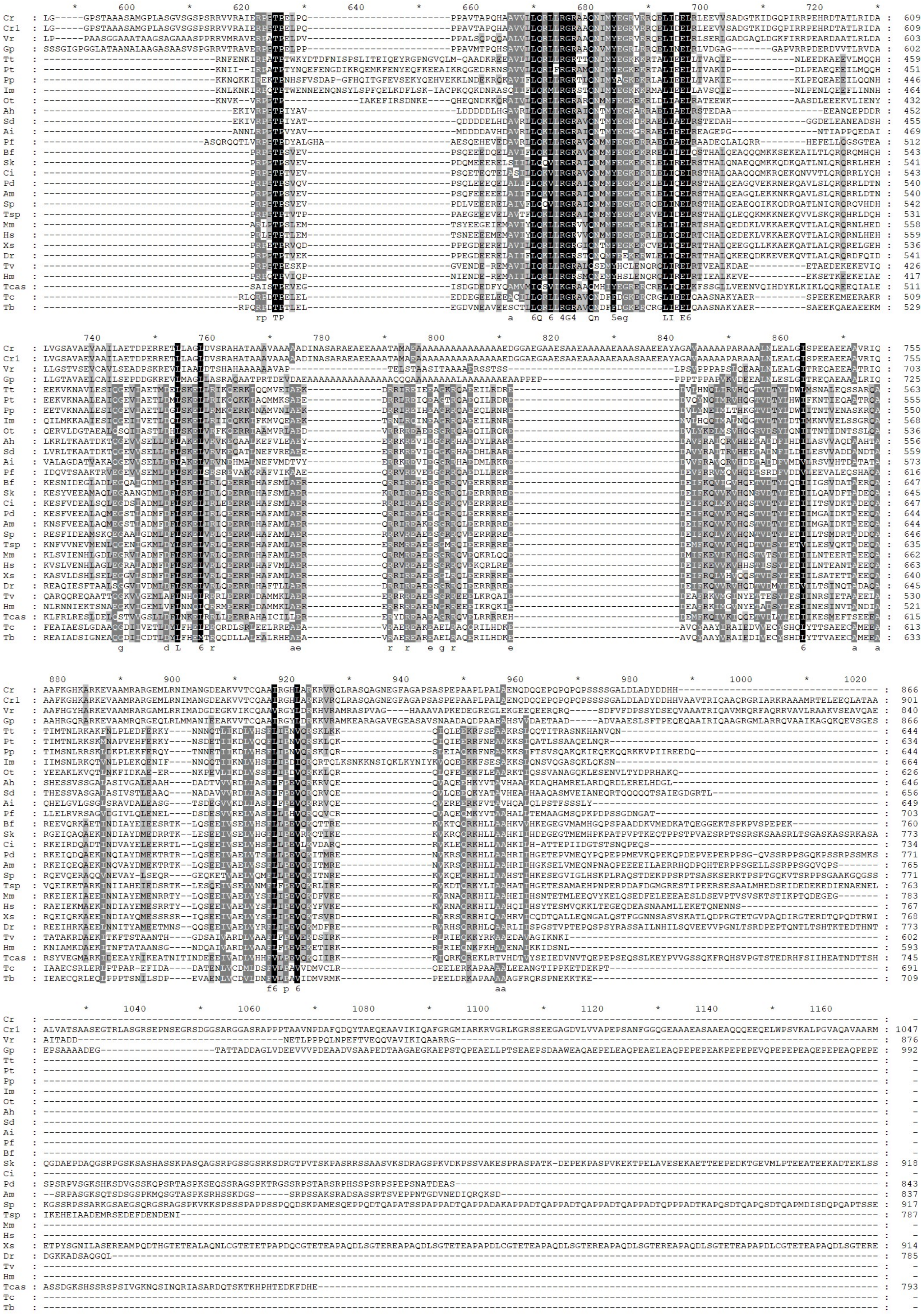

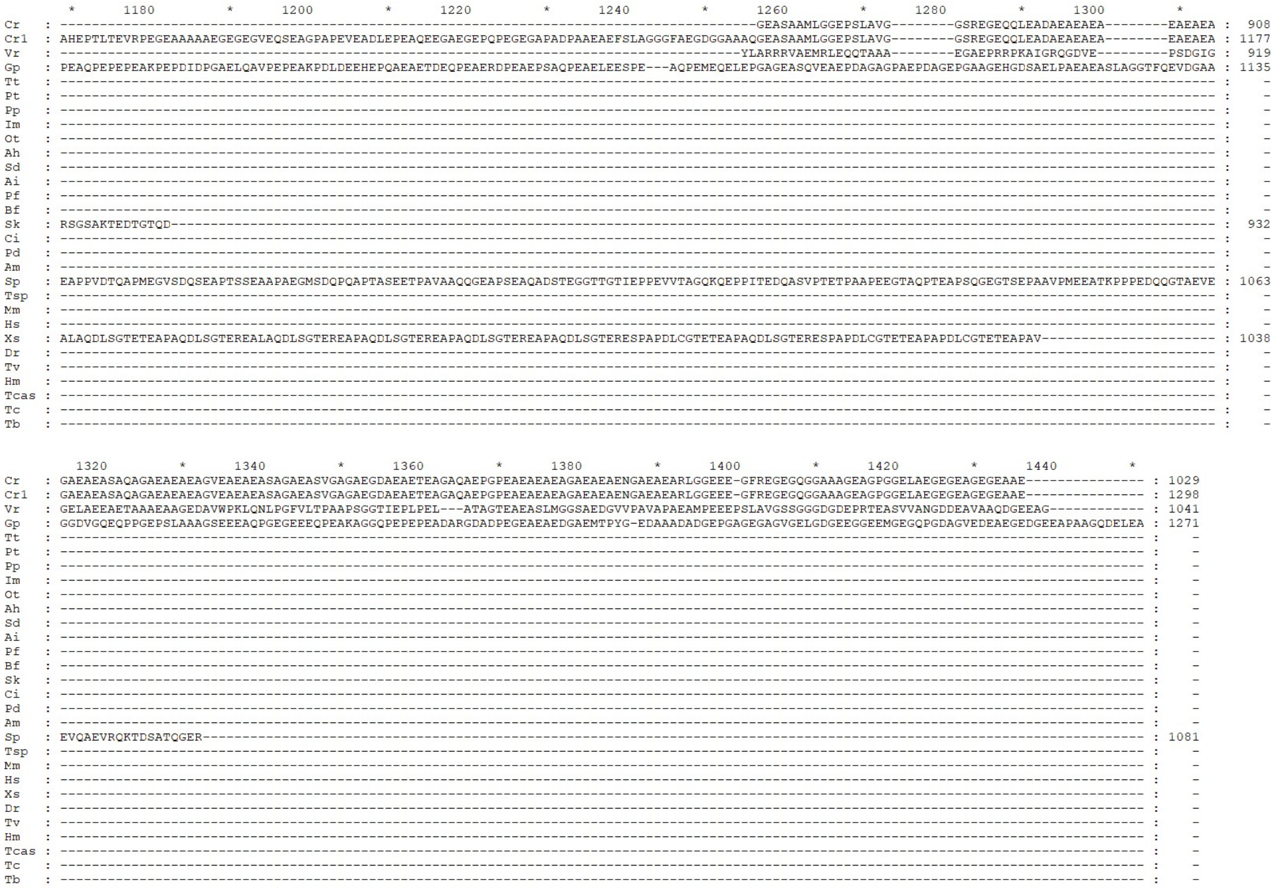

Amino acid sequences of Cfap91 orthologs were obtained from the NCBI protein database using Blastp search and either human, *Chlamydomonas,* or *Tetrahymena* proteins as baits. Protein amino acid sequences were aligned using ClustalX2 [1] software and edited using SeaView [2]. The identical and similar amino acid residues were shaded using GeneDoc [3]. Note that the amino acid sequences of *Chlamydomonas reinhardtii* and two closely related species, *Volvox reticuliferus* and *Gonium pectorale* diverge in their middle and C-terminal fragments compared to Cfap91 orthologs in species from other eukaryotic kingdoms.

Abbreviations with accession number and phylum.

Acropora millepora (Am, XP_044180443.1), Cnidaria; *Achlya hypogyna* (Ah, OQR84847.1), Oomycota; *Aphanomyces invadans* (Ai, XP_008875687.1), Oomycota; *Aureococcus anophagefferens* (Aa, KAH8086166.1), Ochrophyta; *Branchiostoma floridae* (Bf, XP_002606021.1), Chordata; *Chlamydomonas reinhardtii* (Cr, XP_001690436.1, Cr1, XP_042922749.1), Viridiplantae; *Ciona intestinalis* (Ci, XP_002131776.1), Chordata; *Danio rerio* (Dr, XP_001333344.3), Chordata; *Gonium pectorale* (Gp, KXZ53953.1), Viridiplantae; *Histomonas meleagridis* (Hm, KAH0791917.1), Metamonada; *Homo sapiens* (Hs, NP_203528.2), Chordata; *Ichthyophthirius multifiliis* (Im, XP_004027413.1), Ciliophora; *Mus musculus* (Mm, NP_001074494.1), Chordata; *Oxytricha trifallax* (Ot, EJY84328.1), Ciliophora; *Paramecium tetraurelia* (Pt, XP_001449381.1), Ciliophora; *Phytophthora fragariae* (Pf, KAE8886664.1), Oomycota; *Pseudocohnilembus persalinus* (Pp, KRX03757.1), Ciliophora; *Pocillopora damicornis* (Pd, XP_027040548.1), Cnidaria; *Saccoglossus kowalevskii* (Sk, XP_002733402.1), Hemichordata; *Saprolegnia diclina* VS20 (Sd, XP_008605508.1), Oomycota; *Strongylocentrotus purpuratus* (Sp, XP_030833395.1), Echinodermata; *Tetrahymena thermophila* (Tt, XP_001022857.1, TTHERM_00578560), Ciliophora; *Tribolium castaneum*, (Tcas, XP_967867.2), Arthropoda ; *Trichomonas vaginalis G3* (Tv, XP_001312434.1), Metamonada; *Trichoplax sp*. H2 (Tsp, RDD46039.1), Placozoa; *Trypanosoma brucei gambiense DAL972* (Tb, CBH17192.1), Euglenozoa; *Trypanosoma cruzi strain CL Brener* (Tc, XP_818207.1), Euglenozoa; *Volvox reticuliferus* (Vr, GIM05961.1), Viridiplantae; *Xenopus tropicalis* (Xt, NP_001120893.1), Chordata.

References

1. Jeanmougin F., Thompson J.D., Gouy M., Higgins D.G., Gibson T.J. Multiple sequence alignment with Clustal X. *Trends Biochem. Sci.* 1998;23:403–405. doi: 10.1016/S0968-0004(98)01285-7.

2. Galtier N., Gouy M., Gautier C. SEAVIEW and PHYLO_WIN: Two graphic tools for sequence alignment and molecular phylogeny. *Comput. Appl. Biosci.* 1996;12:543–548. doi: 10.1093/bioinformatics/12.6.543.

3. Nicholas K.B., Nicholas H.B. GeneDoc: analysis and visualization of genetic variation. *Embnew. News.* 1997;**4**:14.

Figure S2. Analysis of cilia beating in CFAP91-KO cells. Cilia in the anterior cell end beat in a more coordinated manner and with a slightly higher beat frequency than cilia in the posterior cell end. Upper image: a *Tetrahymena* CFAP91-KO mutant cell with marked oral apparatus (OA) and regions (red lines 1-5) were cilia beating frequency was analyzed. Below (1-5) are kymographs recorded for 0.5 sec (450 frames) and analyzed using in ImageJ program. Note that cilia on both dorsal (2, 3) and ventral (4, 5) cell sides beat with a lower frequency and less regularly than cilia at the anterior cell end (1), with the most apparent alterations near the posterior cell end (3, 5) where the line tracing a cilium movement is nearly flat.

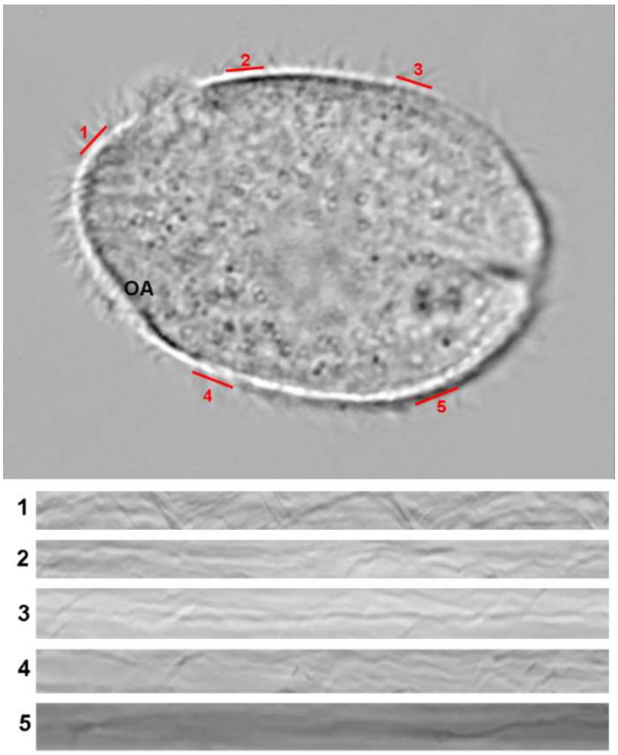

Figure S3. **Lack of Cfap61, Cfap206, or Cfap251 causes reduction of cilia beating frequency.** Examples of kymographs of cilia motility recorded for 0.5 sec (450 frames) and analyzed using ImageJ program. Kymograph of WT cell cilia beating is the same as presented in Figure 4C.

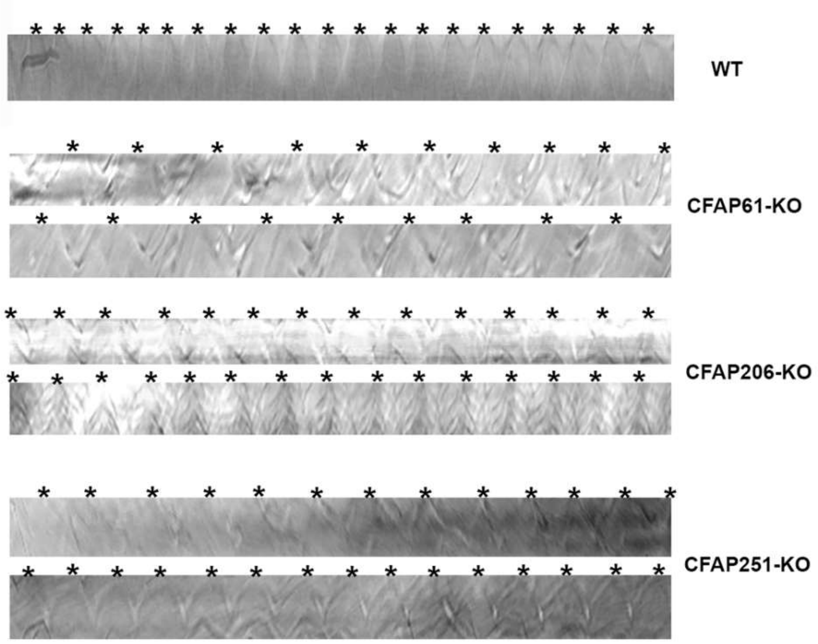

Figure S4. Whole blots used to prepare Figure 8B.

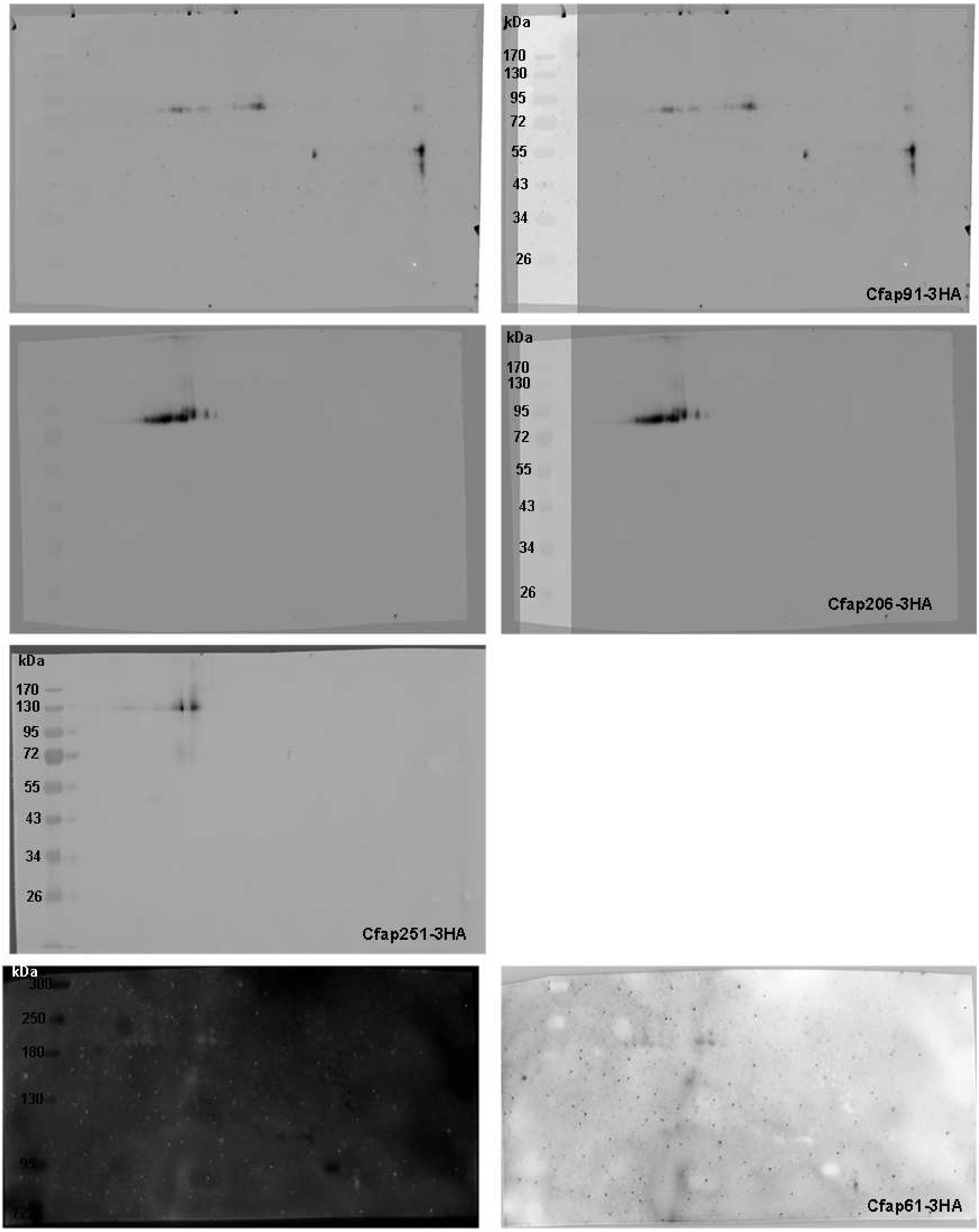

Table S1. Primers used to amplified coding region of analyzed genes.

gene	Primers' nucleotide sequence
CFAP61-ORF-F-nat-MluI	AATT **ACGCGT** GCTTGGACTTAGGATATCTTACAGA
CFAP61 -ORF-R-nat-BamHI	AATT **GGATCC** ATCTACATTTACCTTCTTAGGAGGTACATA
CFAP61-3'UTR-F-PstI	AATT **CTGCAG** CAAACTAAACATGTTACTCCTATTGTT
CFAP61-3'UTR-R-XhoI	AATT **CTCGAG** CATTCAATACTTAACAGGAGAAATCTTC
CFAP91-ORF-F-nat-MluI	AATT **ACGCGT** GTCCTGCAACTCCTACTTG
CFAP91 -ORF-R-nat-BamHI	AATT **GGATCC** ATTCTAAACATTTGCGTGCTTATTTG
CFAP91-3'UTR-F-PstI	AATT **CTGCAG** GAGGAGTAAGTAACCAACAAACC
CFAP91-3'UTR-R-XhoI	AATT **CTCGAG** TCTCTAATTCTAAATTCCAGCTTTCAG
CFAP206-ORF-F-nat-MluI	AAAT **ACGCGT** GTTTATTGCTACCAGGTAAACCT
CFAP206 -ORF-R-nat-BamHI	AATT **GGATCC** ATTAGTGTCTTTGTCTCTTAATCCAGT
CFAP206-3'UTR-F-PstI	AATT **CTCGAG** GGCTAAGATGGAATTATTAAAACTGTAATC
CFAP206 3-3'UTR-R-XhoI	AATT **CTCGAG** AAGCTTATTACAGTTTTAATAATTCCATCTTAGCC
CFAP251-ORF-F-nat-MluI	AATT **ACGCGT** AGCCAATCCAAATGAAGACC
CFAP251 -ORF-R-nat-BamHI	AATT **GGATCC** ATCTTAATCATATTCTTCATTTTCTTCATCTT C
CFAP251-3'UTR-F-PstI	AAAT **CTGCAG** GTAAGTTGCTTACTTGCTTGC
CFAP251-3'UTR-R-XhoI	AATT **CTCGAG** AAGCTTGCAACAGAAATTAAAAACTCACAAAT AATC
calmodulin	Fw: 5' AATT ACGCGT T **ATG**GCTG ATCAAT TAACTG AAGAAC 3'
	Rv: 5' AATT GGATCC **TCA**CTTAG CCATCA TCATTC TAAC 3'
CCDC39	Fw: 5' AATT ACGCGT T **ATG**GATTAGGGAGAATTTATTCAGGA 3'
	Rv: 5' AATT GGATCC **TCA**TTAGTTAGAGATGTTTGATCTTTATGATCTTTGAGAGCG 3'

Supplementary Movie S1. Cilia beating in wild-type cell. Playback speed 25 fps.

Supplementary Movie S2. Cilia beating in CFAP91-KO cell. Playback speed 25 fps.
